# The *Chlamydia trachomatis* Early Effector Tarp Outcompetes Fascin in Forming F-Actin Bundles *In Vivo*

**DOI:** 10.3389/fcimb.2022.811407

**Published:** 2022-03-01

**Authors:** George F. Aranjuez, Jongeon Kim, Travis J. Jewett

**Affiliations:** Division of Immunity and Pathogenesis, Burnett School of Biomedical Sciences, University of Central Florida College of Medicine, Orlando, FL, United States

**Keywords:** *Chlamydia*, tarp, *Drosophila*, F-actin, actin bundles, Singed, Fascin

## Abstract

The intracellular pathogen *Chlamydia trachomatis* secretes multiple early effectors into the host cell to promote invasion. A key early effector during host cell entry, Tarp (translocated actin-recruiting phosphoprotein) is comprised of multiple protein domains known to have roles in cell signaling, G-actin nucleation and F-actin bundle formation. *In vitro*, the actin bundles generated by Tarp are uncharacteristically flexible, however, *in vivo*, the biological significance of Tarp-mediated actin bundles remains unknown. We hypothesize that Tarp’s ability to generate unique actin bundles, in part, facilitates chlamydial entry into epithelial cells. To study the *in vivo* interaction between Tarp and F-actin, we transgenically expressed Tarp in *Drosophila melanogaster* tissues. Tarp expressed in *Drosophila* is phosphorylated and forms F-actin-enriched aggregates in tissues. To gain insight into the significance of Tarp actin bundles *in vivo*, we utilized the well-characterized model system of mechanosensory bristle development in *Drosophila melanogaster*. Tarp expression in wild type flies produced curved bristles, indicating a perturbation in F-actin dynamics during bristle development. Two F-actin bundlers, Singed/Fascin and Forked/Espin, are important for normal bristle shape. Surprisingly, Tarp expression in the bristles displaced Singed/Fascin away from F-actin bundles. Tarp’s competitive behavior against Fascin during F-actin bundling was confirmed *in vitro*. Loss of either *singed* or *forked* in flies leads to highly deformed bristles. Strikingly, Tarp partially rescued the loss of *singed*, reducing the severity of the bristle morphology defect. This work provides *in vivo* confirmation of Tarp’s F-actin bundling activity and further uncovers a competitive behavior against the host bundler Singed/Fascin during bundle assembly. Also, we demonstrate the utility of *Drosophila melanogaster* as an *in vivo* cell biological platform to study bacterial effector function.

## Introduction

Chlamydia is the most commonly reported sexually transmitted bacterial infection in the United States, with an estimated four million cases in the United States in 2018 (Kreisel et al., 2021). Without treatment, Chlamydia infection can lead to pelvic inflammatory disease, which can ultimately lead to permanent damage to the reproductive system. Additionally, infections in pregnant women can also be passed onto the newborn baby, which can lead to eye or lung infections (Mårdh, 2002).

*Chlamydia trachomatis* (*C.t.*) is an obligate intracellular pathogen. Host cell entry, therefore, is a key step in its developmental cycle. The infectious stage of *C.t.*, the elementary body (EB), is equipped with a type III secretion system that injects multiple chlamydial proteins, called early effectors, that facilitate host cell invasion and entry (Peters et al., 2007; Betts et al., 2009). Intracellular *C.t.* reside in a parasitophorous vacuole called an inclusion which cloaks the EB as it differentiates into an actively growing and replicating reticulate body (RB) (Moulder, 1991). Finally, RBs differentiate into infectious EBs and are released to the extracellular space to establish new infections, either through lysis or extrusion of the inclusion (Hybiske and Stephens, 2007).

One of the most well-characterized *Chlamydia trachomatis* early effector is **t**ranslocated **a**ctin-**r**ecruiting **p**hosphoprotein, Tarp. The secretion of Tarp into the host cell is closely followed by F-actin accumulation at the nascent site of EB entry (Clifton et al., 2004). Biochemical studies show that Tarp can directly promote polymerization of actin subunits into filaments, mediated by its C-terminal region (Jewett et al., 2006). Also, blocking the actin binding domain of Tarp inhibits *C. trachomatis* invasion in tissue culture (Jewett et al., 2010). These findings indicate that Tarp’s ability to reorganize the host actin cytoskeleton is a key molecular function required for efficient chlamydial invasion (Jewett et al., 2006; Ghosh et al., 2020).

F-actin can organize into bundles of parallel filaments *via* the crosslinking action of actin bundling proteins. F-actin bundle formation is one mechanism used by cells to exert force on the cell surface to create structures such as filopodia, microvilli, and other membrane protrusions (Stricker et al., 2010). In addition to promoting actin filament formation, Tarp can also directly assemble actin filaments into bundles *in vitro* (Jiwani et al., 2013). However, the physical stiffness of Tarp-assembled bundles deviates from that of bundles assembled by endogenous actin-bundling proteins such as Fascin and alpha-actinin (Ghosh et al., 2018), suggesting that Tarp-mediated F-actin bundles may harbor unique functions in the cell.

Adult *Drosophila melanogaster* are covered in small and large mechanosensory bristles (microchaetes and macrochaetes, respectively). Bristles are made up of chitin that are deposited along long, cellular protrusions originating from bristle cells. These cellular protrusions are generated, in part, by actin polymerization into filaments which arrange into tight, longitudinal bundles arrayed around the periphery of the protrusion (Tilney et al., 1995; Tilney et al., 1996). Defects in F-actin polymerization and bundling impacts the growth of these cellular protrusions, which then manifests as bristle morphology defects (Cant et al., 1994; Petersen et al., 1994; Verheyen and Cooley, 1994; Wu et al., 2016), easily observable in the adult fly.

Two F-actin bundlers, Singed/Fascin and Forked/Espin, are critical for normal bristle shape (Cant et al., 1994; Petersen et al., 1994; Tilney et al., 1995; Tilney et al., 1998). Both proteins participate in the proper formation of tight, longitudinally arrayed F-actin bundles that support the growth of bristle cell protrusions. The absence of either one produces dramatically short and highly malformed bristles (Tilney et al., 1995).

Though Tarp has been associated with actin polymerization during chlamydial invasion, its F-actin bundling properties are newly described and have not been examined *in vivo*. Understanding Tarp-generated F-actin bundles in a physiological context is highly relevant to the greater understanding of the molecular mechanisms of Tarp-mediated chlamydial entry. To achieve this, we utilized *Drosophila melanogaster* as a cell biology platform to study Tarp’s influence on host actin dynamics in an *in vivo* context. Host cell changes due to transgenic expression of Tarp in *Drosophila* tissues can be directly linked to Tarp function without the complex host cell response to active infection. We used the well-characterized development of actin-dependent mechanosensory bristles in *Drosophila* as a platform to understand how Tarp influences actin dynamics in living organisms.

## Materials and Methods

### Generation of Transgenic Flies, Fly Stocks, Crosses, Handling and Rearing

The complete open reading frame of *Chlamydia trachomatis* (serovar L2 strain 434/Bu)(ATCC VR-902B) *Tarp* (Genbank AAT47185.1) and *TmeA* (Genbank AM884176.1, CTL0063), respectively, was cloned into the *Drosophila* transformation and expression vector pUAST (Addgene) (Brand and Perrimon, 1993). The sequence-verified pUAST-Tarp and pUAST-TmeA plasmids were sent for injection into *D. melanogaster w^1118^* embryos (Model System Injections, North Carolina).

The following *D. melanogaster* stocks were kind gifts from J. McDonald (Kansas State University) or obtained from the Bloomington *Drosophila* Stock Center (stock number in parentheses): yw;ap-GAL4/CyO (J. McDonald), hs-GAL4(II) (2077), hs-GAL4(III) (1799), w;pnr-GAL4/TM3,Ser,UAS-y (3039), w*;ubi-GAL4/CyO (32551), hs-FLP;Act>y+>GAL4,UAS-mCD8:GFP/CyO (J. McDonald), y,w1118;UAS-EGFP5a.2 (5431), y,sc,v;UAS-*forked* RNAi,y+ (41678), y;UAS-s*inged* RNAi,y+ (57805). The following stocks were made to test the ability of Tarp to rescue loss of endogenous bundlers: ap-GAL4, UAS-*singed* RNAi/CyO and pnr-GAL4, UAS-*forked* RNAi, y+. Rescue crosses were kept at 22°C.

The FLPout-GAL4 system (Blair, 2003) was used to generate clonal populations of follicle epithelial cells. Tarp-expressing clones were generated by crossing hs-FLP;Act>y+>GAL4,UAS-mCD8:GFP/CyO (J. McDonald) to UAS-Tarp, and the desired adult progeny subjected to heat shock in a 37°C water bath for 1 hour. The flies were kept at 25°C for 1 day prior to dissection.

Unless otherwise indicated, all fly stocks and crosses were reared at 25°C on Nutri-fly BF media (Genesee Scientific) supplemented with 0.45% v/v propionic acid.

### Immunostaining and Confocal Microscopy

Individual ovarioles from *Drosophila* ovaries were dissected as described previously (McDonald and Montell, 2005). Ovarioles were fixed in 4% methanol-free formaldehyde (Thermo) in phosphate-buffered saline with 0.1% v/v Triton-X-100 (PBT) for 10 minutes with rocking. Fixed ovarioles were subsequently blocked with 0.5% w/v bovine serum albumin in PBT for 2 hours. GFP expression marks clonal populations of follicles with active GAL4/UAS expression.

For pupal bristle imaging, pupae were collected at 36-50 hours post-puparium formation—the stage where bristle primordia are actively growing from bristle cells in the dorsal thorax. The dorsal pupal casing was removed, and the dorsal pelt dissected using microscissors and fine dissecting forceps. The dorsal pelt was fixed in 4% methanol-free formaldehyde in PBT at room temperature for 10 minutes and blocked with 0.5% w/v bovine serum albumin in PBT for 2 hours.

The following antibodies were used for immunostaining: mouse anti-Singed, 1:100 (DSHB, sn 7c); rabbit polyclonal anti-Tarp, 1:1000 (Clifton et al., 2004); mouse monoclonal anti-phosphotyrosine (4G10, Millipore-Sigma). F-actin was visualized using Alexa 647-conjugated phalloidin (Molecular Probes, 1:400). The appropriate Alexa-conjugated secondary antibodies (Alexa 350, Alexa 568, Alexa 647, Molecular Probes) were used at 1:400. Immunostained tissue were mounted under a #1.5 cover glass using Aqua-Polymount mounting medium (Polysciences).

A Zeiss LSM 710 confocal microscope was used to acquire fluorescent images of follicle cells using a 40X Plan-Neuofluar oil objective (epithelial follicle cells) or a 100X Plan-Apochromat oil objective (pupal bristle cells). Image acquisition settings and minor adjustments to brightness and contrast post-acquisition were identical between control and Tarp-expressing follicle cells.

### Imaging Adult *Drosophila* Bristles

Adult flies, with wings removed, were glued onto wooden picks. The dorsal thorax was imaged using a Zeiss Stemi 508 stereomicroscope equipped with an Axiocam 208 color camera. Minor brightness and contrast adjustments were made in Adobe Photoshop.

### Scoring Bristle Morphology Defects

The dorsocentral and scutellar bristles of adult flies were examined under a dissecting stereomicroscope. The following scores were assigned: ‘0’: wild-type bristle—oriented towards the posterior direction, with a gentle, uniform curve, and tapering thickness towards the tip; ‘1’: bristle with an increased curvature, but no sharp bends or kinks; ‘2’: bends or kinks are present but the angle is around 45°; ‘3’: bends or kinks are at approximately 90°; ‘4’: bends or kinks are greater than 90°, multiple bends or kinks are often observed. Pairs of dorsocentral or scutellar bristles were scored and summed up, allowing each fly to get a minimum score of 0 up to a highest possible score of 16.

### Actin Bundling Sedimentation Assay

Briefly, actin filaments were generated by adding 1/10 volume of actin polymerization buffer (500mM KCl, 20mM MgCl_2_, 10mM ATP, in 100mM Tris pH 7.5) to reconstituted monomeric rabbit actin (0.5mg/mL, Cytoskeleton Inc) followed by a 1-hour room temperature incubation. Approximately 5 µg of purified Tarp, Fascin, BSA or a combination of Tarp (1 to 5µg) and Fascin (added simultaneously or sequentially) was added to 40 µg of F-actin and allowed to incubate for 1 hour at room temperature. Actin bundles and bound proteins were separated by differential sedimentation at 10,000 X *g* for 1 hour in a Beckman Optima MAX-TL Ultracentrifuge using a TLA55 or TLA 100.3 rotor (Beckman Coulter, Life Sciences). Proteins associated with the actin bundles in the pellet were compared to unbound proteins that remained in the supernatant by resolving the proteins on 4-12% SDS-polyacrylamide gels followed by Coomassie staining. Densitometric analysis of protein bands were performed in FIJI (Schindelin et al., 2012).

### TmeA Antibody Generation and Western Blotting

The complete open reading frame of L2 TmeA (CT694, CTL0063) was cloned into pGEX-6p-1 (Cytiva Life Sciences) and expressed as a GST-fusion. The fusion protein was purified with glutathione sepharose beads and eluted from the column following digestion with PreScission Protease according to the directions of the GST-Bulk kit (Cytiva Life Sciences). The GST-free TmeA was used an immunogen to produce specific guinea pig polyclonal antibodies (Cocalico Biologicals Inc.).

For heat shock induction, fly vials were placed in a 37°C water bath for 1 hour, and immediately homogenized in SDS-PAGE sample buffer. Whole flies were crushed in SDS-PAGE sample buffer (10 flies per 100 µl sample) and heated at 95°C for 10 minutes. Samples were run on 4-12% SDS-polyacrylamide gels. The following antibodies were used: rabbit polyclonal α-Tarp, 1:1000 (Clifton et al., 2004); guinea pig polyclonal α-TmeA (1:50, affinity purified); mouse monoclonal α-GAPDH (Invitrogen, GA1R); mouse monoclonal α-β-actin antibody (BD Biosciences, C4); anti-rabbit and anti-mouse HRP-conjugated antibodies; 1:10,000 (Millipore). Immunoblots were imaged on a Bio-Rad ChemiDoc Imager.

### Graphs, Figures, and Statistics

Graphpad Prism was used to generate graphs and perform statistical tests. Figures were generated and assembled using FigureJ (Mutterer and Zinck, 2013), FIJI (Schindelin et al., 2012) and Adobe Illustrator.

## Results

### Tarp Is Phosphorylated and Forms F-Actin Aggregates in *Drosophila melanogaster*

**Figure 1:**
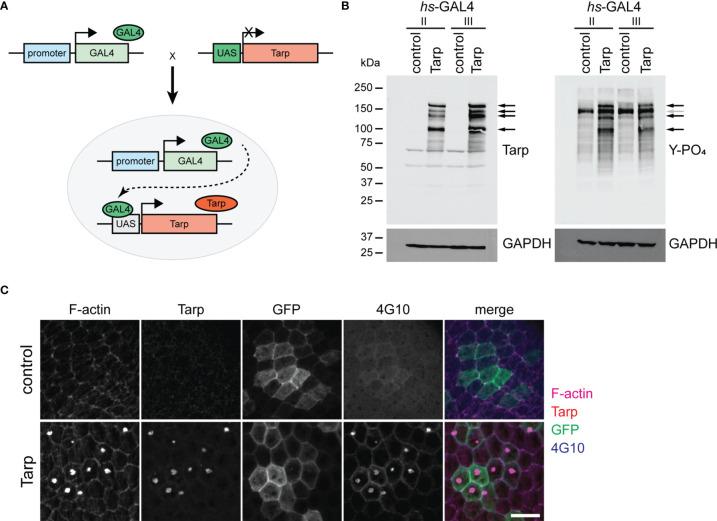
Transgenically expressed Tarp is phosphorylated and forms actin-rich aggregates in flies. **(A)** Schematic diagram of the GAL4/UAS binary expression system. A transgenic fly stock was created, containing the Tarp open reading frame downstream of the UAS promoter. The Tarp transgenic fly is crossed with another fly that encodes for the GAL4 transcription factor with a defined expression pattern. The progeny of the cross combines both components, prompting Tarp expression in cells where GAL4 is expressed. **(B)** Western blots showing Tarp expression and corresponding tyrosine phosphorylation in flies. Black arrows indicate Tarp bands. Gray arrow indicates the location of a Tarp band obscured by endogenous phosphotyrosine signal. hs-GAL4/UAS-Tarp flies were heat-shocked at 37°C for 1 hour. hs-GAL4/+ flies were used as control. Whole fly lysates were analyzed for the expression of Tarp (left) and tyrosine phosphorylation (right). Two unique hs-GAL4 lines were used (II and III). GAPDH was used as loading control. **(C)** Tarp localizes to actin-rich aggregates in epithelial cells. Clonal populations of follicle epithelial cells (see Immunostaining methods) were immunostained for Tarp, F-actin, and tyrosine phosphorylation (4G10). GFP identifies cells in which GAL4/UAS expression is active. Scale bar,10 μm.

We generated transgenic *Drosophila melanogaster* engineered to express *Chlamydia trachomatis* L2 Tarp. Expression was temporally and spatially controlled in various tissues of interest using the GAL4-UAS binary expression strategy (Brand and Perrimon, 1993). To drive Tarp expression, UAS-Tarp flies were mated (a.k.a. crossed) with unique GAL4 flies resulting in progeny capable of tissue-specific Tarp expression (**Figure 1A**).

To verify the expression of Tarp in flies, we ubiquitously expressed Tarp in all tissues using a heat shock-inducible GAL4. Inducible expression allows for temporal control of Tarp expression and bypasses potential toxicity defects that may halt the developmental cycle prior to the adult stage. Western blot analysis shows robust Tarp expression in hs-GAL4/UAS-Tarp flies (Tarp) (**Figure 1B**, left, arrows). We observed bands corresponding to full-length Tarp (>150kDa) as well as possible proteolytic cleavage products. Whole fly lysates from hs-GAL4/UAS-GFP flies served as the negative control.

During chlamydial invasion, Tarp is rapidly phosphorylated in the host cell following translocation *via* type III secretion apparatus (Clifton et al., 2004). We tested whether transgenically expressed Tarp is similarly phosphorylated in *Drosophila*. Western blot analysis revealed a collection of phosphotyrosine-rich proteins whose migration coincided with nearly all Tarp-specific bands (**Figure 1B**, right, black arrows). Control fly lysates revealed a single prominent phosphotyrosine band at ~150 kDa, likely obscuring one of the Tarp-corresponding bands (**Figure 1B**, right, gray arrow). This correlation provides further evidence of Tarp expression in flies and shows that Tarp can be phosphorylated by endogenous *D. melanogaster* kinases.

We also examined the cellular localization of Tarp in *Drosophila* cells by driving expression in epithelial follicle cells of the egg chamber. The egg chamber, the basic subunit of *Drosophila* oogenesis, is completely enveloped by a monolayer of epithelial follicle cells and are highly amenable to dissection and immunofluorescence staining without disrupting tissue organization. Clonal populations of follicle cells that drive ubiquitous transgene expression were randomly generated (see *Materials and Methods*). These clonal follicle cell populations are marked by GFP expression. Tarp expression resulted in the formation of F-actin aggregates and Tarp itself was enriched in said aggregates (**Figure 1C**, bottom). Moreover, these aggregates were enriched for tyrosine phosphorylation (**Figure 1C**, bottom). Control follicle cells that expressed GFP alone had no detectable F-actin aggregates (**Figure 1C**, top).

Collectively, these results demonstrated that transgenically expressed Tarp was recognized by endogenous kinases in *Drosophila*. Also, the ability of Tarp to modify F-actin was preserved in the cellular environment of *Drosophila* cells. This suggests that Tarp interaction with the cellular environment is conserved between *Drosophila* and humans.

### Tarp Induces Abnormal Curvature of the *Drosophila* Mechanosensory Bristles

**Figure 2:**
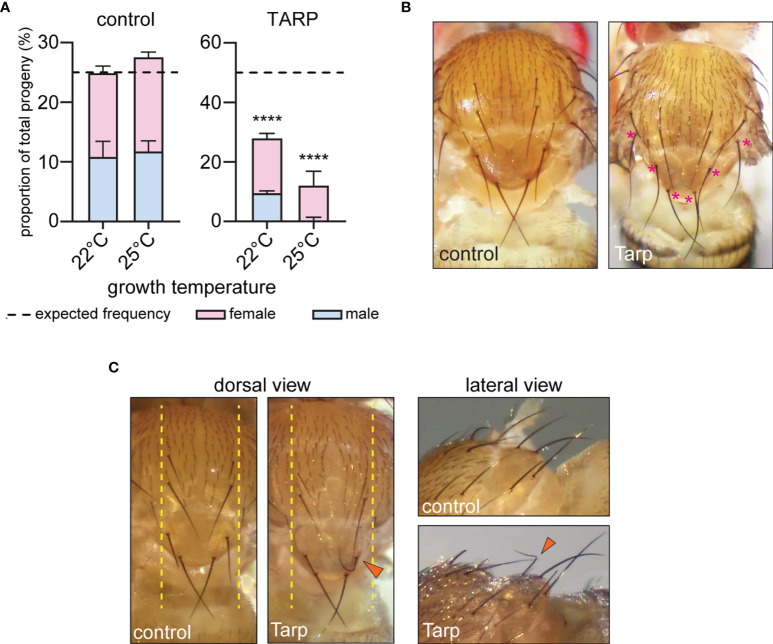
Tarp induces abnormal curvature of large mechanosensory bristles. **(A)** The proportion of ubiquitously expressing GFP (control) or Tarp flies within the progeny population was recorded. The impact of Tarp expression on fly viability was assessed by comparing the observed proportion to expected Mendelian ratio (dashed lines). Fischer’s exact test was used to test for significance between observed and expected values (****p<0.0001). The male-to-female ratio is indicated by blue and pink bars, respectively. **(B)** Dorsal thorax of adult flies ubiquitously expressing GFP (control) or Tarp, displaying small and large mechanosensory bristles. In Tarp-expressing flies, asterisks (pink) mark large mechanosensory bristles that display abnormally increased curvature. **(C)** Dorsal thorax of flies that express GFP (control) or Tarp in the central region of the thorax (yellow dashed lines) *via* pnr-GAL4. An abnormally curved bristle (arrowhead) is observed in Tarp-expressing flies. The same flies were imaged in dorsal and lateral view.

We sought to prove Tarp functionality in flies by assessing the impact of the effector on *Drosophila* development. We expressed Tarp constitutively in all tissues using ubi-GAL4, which utilizes the ubiquitin gene *Ubi-p5E* promoter. Tarp expression was validated by Western blot (**Figure S1**). The impact of ubiquitous Tarp expression on viability was assessed by comparing the observed proportion of ubi>Tarp flies within the total progeny population to the expected Mendelian ratio (**Figure S2**). Ubiquitous expression of GFP alone (control) did not impact adult viability and did not skew the male-to-female ratio (**Figure 2A**, left). Conversely, ubiquitous expression of Tarp resulted in a marked decrease in adult survival, recovering about half of the expected Tarp-expressing progeny (**Figure 2A**, right). The male-to-female ratio was also skewed with fewer than expected adult males recovered. GAL4-UAS expression in flies is influenced by temperature, with higher fly rearing temperatures associated with stronger expression (Riabinina et al., 2015). Consistent with this, we observed an enhancement of adult lethality upon ubiquitous Tarp expression when flies are reared at slightly higher temperatures (22°C vs. 25°C) (**Figure 2A**).

To test the specificity of the Tarp-induced lethality, we tested transgenic fly lines that can express the *C.t.* early effector TmeA. Similar to Tarp, TmeA is important for host cell invasion by directing cytoskeleton changes *via* altered Arp2/3 dynamics (McKuen et al., 2017; Faris et al., 2020; Keb et al., 2021). Expression of TmeA in flies *via* the ubi-GAL4 driver was validated by Western blot (**Figure S3A**). Importantly, there was no difference observed in the fly viability upon ubiquitous expression of TmeA compared to GFP (**Figure S3B**).

These findings demonstrate that ubiquitous expression of Tarp in *Drosophila* perturb critical aspects of fly development which manifest as reduced adult survival. From here, we then focused on fly tissues that are sensitive to changes in F-actin dynamics to study Tarp’s influence on host actin.

A closer inspection of surviving adult flies that ubiquitously expressed Tarp revealed morphological changes in the large mechanosensory bristles, or macrochaetes, of the dorsal thorax. During pupal development, bristle cells extend a single, cellular protrusion, driven and stabilized by the assembly of large, ordered arrays of F-actin bundles (Tilney and DeRosier, 2005). Macrochaetes of control flies appeared uniformly straight from the dorsal view (**Figure 2B**, left). In contrast, macrochaetes with pronounced curvatures were observed in ubi>Tarp flies examined, particularly the posterior bristles of the thorax (**Figure 2B**, asterisks).

To precisely characterize the observed Tarp-induced bristle curvature, we expressed Tarp in a more defined spatial pattern using a different GAL4 driver, pnr-GAL4, whose expression pattern is restricted to tissues along the dorsal midline (Mummery-Widmer et al., 2009) (**Figure 2C**, dashed lines). Control flies that expressed GFP along the midline had normal macrochaete morphology (**Figure 2C**)(**Table 1**). Consistent with the phenotype of flies ubiquitously expressing Tarp, highly curved macrochaetes were observed for flies expressing Tarp along the midline (**Figure 2C**, arrowhead)(**Table 1**). One third of pnr-GAL4>UAS-Tarp flies (37% of males, 31% of females)(**Table 1**) displayed highly curved macrochaetes and, in most cases, only one macrochaete was visibly affected per fly. Bristle morphology defects were not observed when the *C.t.* effector TmeA is expressed in the thorax (**Figure S3C**), showing Tarp specificity of the phenotypes observed.

**Table 1:** Frequency of bristle phenotypes in control and Tarp-expressing flies using pnr-GAL4.

bristle phenotypes	males								females							
	pnr>GFP				pnr>Tarp				pnr>GFP				pnr>Tarp			
	trial 1	trial 2	trial 3	Ave	trial 1	trial 2	trial 3	Ave	trial 1	trial 2	trial 3	Ave	trial 1	trial 2	trial 3	Ave
scutellar bristle, curved	0%	0%	0%	0%	35%	36%	39%	37%	0%	0%	0%	0%	30%	29%	33%	31%
scutellar bristle, upturned	0%	0%	0%	0%	7%	0%	18%	8%	0%	0%	0%	0%	5%	8%	6%	6%
scutellar bristle, bent	0%	0%	0%	0%	2%	0%	0%	1%	0%	0%	0%	0%	0%	0%	0%	0%
number of flies scored	30	47	26		55	11	28		41	41	29		56	24	36	

Therefore, the presence of Tarp interferes with fly bristle morphology, which manifests as increased bristle curvature. This is likely due to Tarp’s specific influence on host actin dynamics since bristle shape depends on actin filament formation and bundling.

### Tarp Can Substitute for the Loss of the Endogenous F-Actin Bundler Singed/Fascin During Bristle Development

**Figure 3:**
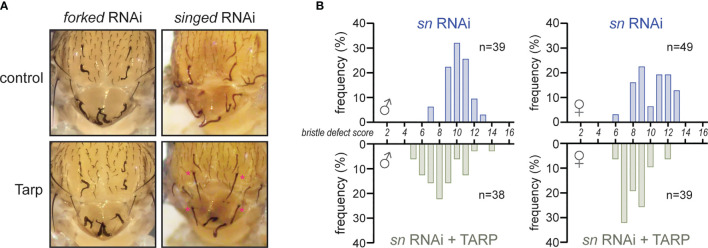
Tarp partially rescues the loss of the host F-actin bundler Singed/Fascin during bristle development. **(A)** RNAi knockdown of host F-actin bundlers *forked* and *singed* result in severe bristle morphology defects. Tarp expression in the *singed* RNAi background restores bristles to near-normal morphology (asterisks). **(B)** Histograms of bristle defect scores of male and female fly populations of *singed* RNAi knockdown alone (blue bars) or with Tarp expression (green bars). Each fly was assigned a bristle defect score: ‘0’ being wildtype; and ‘16’ as complete and severe defect (see *Material and Methods*). There is a leftward shift of bristle defect score distribution in the fly populations that express tarp in the *singed* RNAi background.

Tarp can promote actin filament formation from monomeric actin (Jewett et al., 2006). Interestingly, it also induces the formation of F-actin bundles *in vitro* (Jiwani et al., 2013; Ghosh et al., 2018). We tested the physiological relevance of Tarp’s F-actin bundling property *in vivo* using *Drosophila* bristles as a cell biological model. There are two F-actin bundlers that are required for normal bristle shape: 1) Forked/Espin; and 2) Singed/Fascin (Tilney et al., 1998). The loss of either *forked* or *singed* leads to short bristles with multiple sharp bends or curls (Cant et al., 1994; Petersen et al., 1994). We tested whether Tarp could alleviate the bristle morphology defect in bristles upon knockdown of endogenous bundlers. Engagement of the RNAi machinery *via* the expression of double-stranded RNA targeting GFP in the thorax does not lead to bristle morphology defects (**Figure S4**). On the other hand, RNAi knockdown of *forked* or *singed* in the thorax led to highly abnormal bristles (**Figure 3A**, top row), similar to the corresponding loss-of-function mutations (Cant et al., 1994; Petersen et al., 1994). Tarp expression in the *forked* RNAi background did not relieve the bristle phenotype (**Figure 3A**, bottom left). In contrast, Tarp expression in the *singed* RNAi background resulted in appreciable reduction of the bristle phenotype (**Figure 3A**, bottom right, asterisks).

To better describe the observed rescue of *singed* knockdown, we devised a scoring rubric to systematically appraise and compare the observed bristle morphology defects across different fly populations (see *Material and Methods*). A bristle defect score of ‘0’ represents normal, wild-type bristles while a maximum score of ‘16’ represents severe malformation of all bristles examined. *singed* knockdown flies show a distribution of scores centered around ‘10’ for both males and females, with the females having a slightly larger spread (**Figure 3B**, blue bars). Expression of Tarp in the *singed* RNAi background results in a leftward shift of the peak of the bristle defect score histogram for both male and female populations (**Figure 3B**). This indicates a partial alleviation of the *singed* knockdown phenotype in the Tarp-expressing fly population.

This demonstrates that Tarp can partially take the place of an endogenous F-actin bundler to promote normal bristle morphology, providing physiological evidence of Tarp’s bundling activity.

### Tarp Alters the Localization of the Endogenous F-Actin Bundler Singed in Developing Bristles

**Figure 4:**
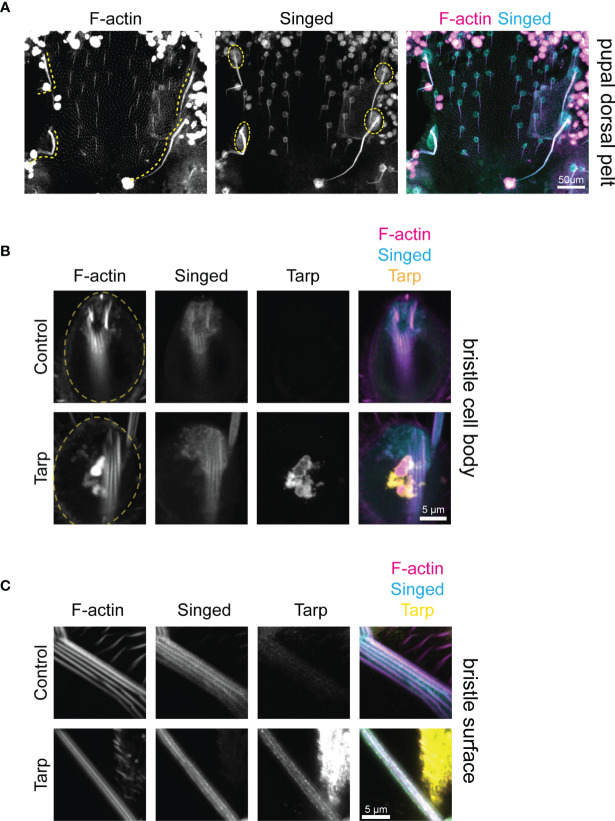
Tarp is found within the growing bristle primordium during pupal development. **(A)** Confocal micrographs of a dissected dorsal pelt of control *Drosophila* pupa immunostained to visualize F-actin and Singed localization. Developing large mechanosensory bristles (yellow dashed lines) emerge from bristle cell bodies (yellow dashed circles). The number and topology of bristles are already determined at the pupal stage. **(B)** High-magnification confocal micrographs of bristle cell bodies (yellow dashed circles) from control or Tarp-expressing pupae. Tarp is enriched in F-actin-abundant aggregates in the bristle cell body. F-actin bundles are found at the base of the bristle primordium. **(C)** Thin optical sections of high-magnification representative confocal micrographs of developing pupal bristles from control (n=9) and Tarp-expressing (n=8) pupae. Longitudinally-arrayed F-actin bundles and Singed localization was observed for both genotypes. Tarp is similarly found within the growing bristle in Tarp-expressing flies. For all panels, Singed and Tarp localization was determined by immunostaining. F-actin was visualized using Alexa-conjugated phalloidin. Tarp expression was driven by pnr-GAL4.

The final shape and size of mechanosensory bristles is established during pupal development before flies hatch. We dissected the dorsal pelt of *Drosophila* pupae to better understand the impact of Tarp expression on bristle cell biology. The elongating bristles of developing macrochaetes are readily visualized using fluorescent phalloidin, highlighting the abundance of F-actin in these structures (**Figure 4A**, left, dashed lines). Singed is also highly enriched in the bristle cells, both in the growing protrusion as well as the bristle cell body (**Figure 4A**, middle, dashed circles). Last, the number and topology of mechanosensory bristles are already established during pupal development. This allows us to identify the specific bristles that exhibit increased curvature in the adult.

Since pnr-GAL4 drives Tarp expression over a broad central region of the thorax made up of different cell types (**Figure S5**), we sought to verify that Tarp is expressed in the developing bristle cells themselves. High-magnification imaging of the cell body of control bristle cells showed F-actin outlining the cell body and enriched at the base of the bristle primordium; Singed is similarly enriched at the bristle primordium base (**Figure 4B**, top row). pnr>Tarp pupal bristle cells appear similarly but with the addition of a striking F-actin-rich Tarp aggregate in the cell body (**Figure 4B**, bottom row). Striped phalloidin staining at the bristle primordium base of both control and Tarp-expressing bristles represent F-actin bundles that extend into the growing bristle primordium.

We further investigated whether Tarp can be found within the growing bristle primordium itself. Control bristles contained longitudinally arranged F-actin bundles (**Figure 4C**, top). Singed was also enriched within growing bristles, in similar longitudinal stripes (**Figure 4C**, top). Bristles from Tarp-expressing cells displayed prominent F-actin bundles as well as longitudinal stripes of Singed enrichment (**Figure 4C**, bottom). In addition, Tarp was detected within the developing bristle itself, enriched in longitudinal stripes but distributed heterogeneously, presenting either as distinct or contiguous clumps (**Figure 4C**, bottom). The large area of Tarp staining outside the bristle comes from surrounding epithelial cells of the pupal pelt. To image distinct F-actin bundles, thin optical sections of the pupal bristle were imaged, which affects the apparent thickness of the bristle. No major difference in pupal bristle thickness was observed between genotypes examined.

**Figure 5:**
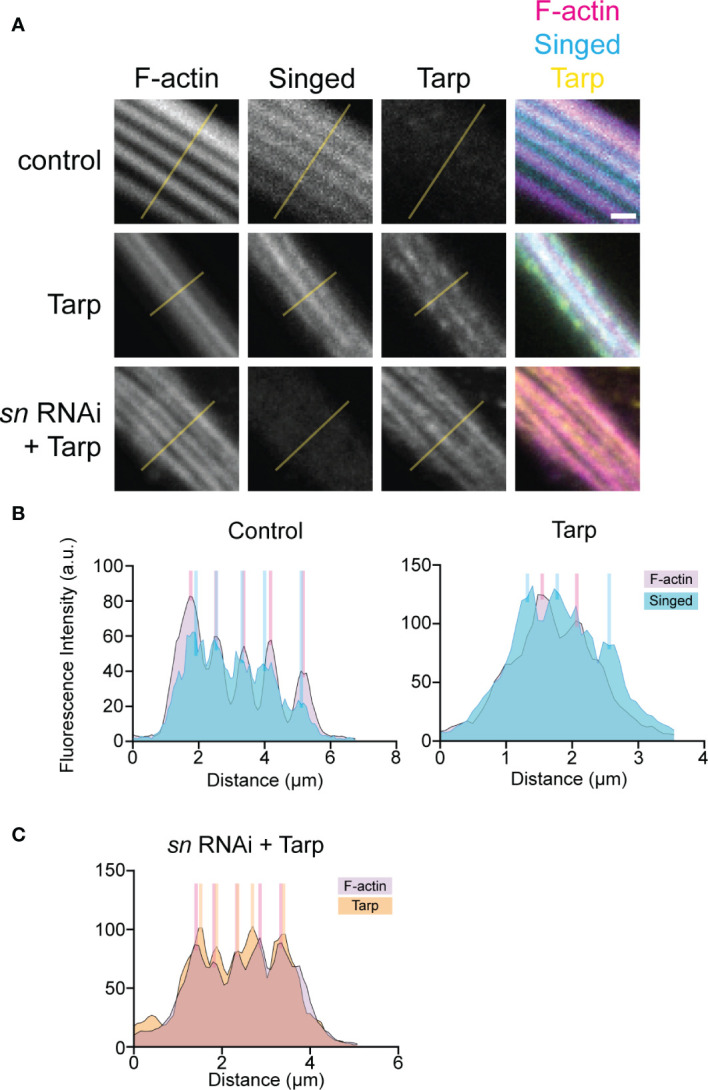
Tarp expression results in mislocalization of host F-actin bundler Singed away from F-actin bundles in developing pupal bristles. **(A)** Line analysis of high-magnification confocal micrographs of control and Tarp-expressing pupal bristles, as well as pupal bristle from flies that express Tarp in the *singed* RNAi background. Fluorescent pixel intensity along the length of the yellow line were plotted for each indicated channel **(B, C)**. Peak fluorescent intensities, corresponding to the localization patterns, were highlighted with colored bars. Scale bar, 1μm. Representative data and images are shown out of three trials.

We performed line intensity analysis on the immunostained bristles to visualize protein localization within the bristles. Control bristles displayed a strong correlation between the F-actin bundles and the endogenous bundler Singed, consistent with its known function (**Figures 5A, B**, left). Surprisingly, when Tarp was expressed in bristles, the peak Singed fluorescence intensity occurs away from the F-actin bundles (**Figures 5A, B**, right). Pupal bristles from flies that simultaneously knockdown the actin bundler *singed* while expressing Tarp shows a strong correlation between F-actin bundles and Tarp (**Figures 5A, C**), consistent with Tarp’s ability to alleviate the *singed* RNAi phenotype.

This observation suggests the possibility of a novel interaction between Tarp and Singed, wherein Tarp may negatively influence the ability of the host F-actin bundler to participate in actin bundle formation.

### Tarp Outcompetes Fascin in Forming F-Actin Bundles *In Vitro*

**Figure 6:**
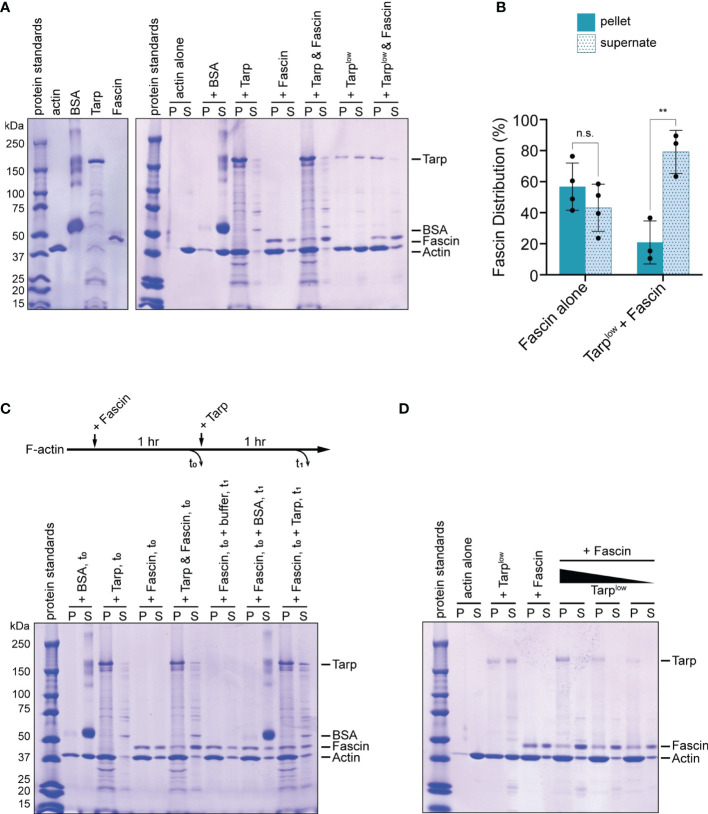
Tarp outcompetes Fascin in F-actin bundling formation *in vitro*. **(A)** An *in vitro* F-actin bundling assay was performed using purified Tarp and Fascin, alone or in combination. Bovine serum albumin (BSA) does not interact with F-actin and serves as negative control. F-actin bundles, together with associated bundling proteins, are enriched in the pellet fraction. In the presence of Tarp or Fascin, actin is enriched in the pellet fraction as well as the corresponding F-actin bundlers. Simultaneous addition of Tarp and Fascin to F-actin results enrichment of actin and Tarp in the pellet fraction, while the majority of Fascin is found in the supernate. Exclusion of Fascin from the pellet fraction still occurs even with a reduced amount of Tarp (Tarp^low^). **(B)** Fascin distribution following F-actin bundling assays when added alone (Fascin alone) or simultaneously with Tarp (Tarp^low^ + Fascin). Multiple trials are represented. Fascin is predominantly found in the supernate fraction when Tarp is present during F-actin bundling (Welch’s t test, **p<0.01). n.s. not significant. **(C)** *In vitro* F-actin bundling was further assessed after sequential addition of Tarp and Fascin. Individual proteins, as well as Tarp and Fascin together, added at the beginning of the assay and sampled at t_0_, recapitulated the original finding. Fascin, followed by the addition of buffer or BSA, at t_0_, resulted in F-actin bundling and enrichment of Fascin in the pellet fraction, upon analysis at t_1_. Subsequent addition of Tarp at t_0_ to Fascin-assembled bundles resulted in enrichment of Tarp in the pellet fraction when, with minimal impact on Fascin distribution, upon analysis at t_1_. **(D)** When added simultaneously, Tarp is able to outcompete Fascin during F-actin bundle formation even with diminishing amounts of Tarp. Individually, Tarp (Tarp^low^) and Fascin efficiently form F-actin bundles and Fascin is highly enriched in the pellet fraction. On the other hand, Fascin is predominantly shifted to the supernate fraction, across all Tarp concentrations tested.

To further investigate the competitive behavior between Tarp and Fascin (Singed), we performed an *in vitro* F-actin bundling assay using purified Tarp and Fascin. Incubation of pre-formed actin filaments with actin bundlers results in the formation of F-actin bundles, which can be isolated *via* low-speed centrifugation. On its own or with bovine serum albumin (BSA) as a negative control, filamentous actin was present primarily in the supernatant, indicating the lack of F-actin bundle formation (**Figure 6A**). Assessed individually, Fascin and Tarp were both potent bundlers of F-actin (**Figure 6A**).

Simultaneous addition of Tarp and Fascin to filamentous actin resulted similarly efficient F-actin bundling, with the vast majority of Tarp found in the pellet fraction (**Figure 6A**). Surprisingly, the converse was true for Fascin, with a majority of Fascin detected in the supernatant fraction (**Figures 6A, B**). Thus, when added simultaneously, Tarp can exclude Fascin from associating with actively assembling F-actin bundles. This suggests that Tarp has a competitive advantage over Fascin in participating in F-actin bundle formation.

As an early effector that is secreted into living cells during infection, beyond driving actin bundle formation *de novo*, Tarp may also interact with existing F-actin bundles. We therefore sought to determine the outcome of Tarp-Fascin dynamics in the presence of pre-assembled actin bundles. To do this, Fascin was added to F-actin and incubated to form bundles (t_0_). Subsequently, buffer, BSA (negative control) or Tarp was added to the samples, followed by a second incubation (t_1_). Buffer treatment or the addition of BSA did not alter Fascin-assembled F-actin bundles nor the enrichment of Fascin in the pellet fractions (**Figure 6C**). Upon addition of Tarp to Fascin-assembled bundles, a large proportion of Tarp was found in the pellet fraction (**Figure 6C**), indicating an association with F-actin bundles. Interestingly, we did not observe a strong Fascin distribution to the supernate in this condition. Instead, Fascin distribution was comparable to that when Fascin is added to F-actin alone. We further examined this competitive behavior by testing different concentrations of Tarp during simultaneous co-incubation with Fascin. The competitive interaction was preserved even with a reduced amount of Tarp protein (Tarp_low_) (**Figure 6A**, last two lanes). More strikingly, Fascin continues to be outcompeted through sequential reduction of Tarp concentration, remaining strongly enriched in the supernate fraction (**Figure 6D**).

## Discussion

*Drosophila melanogaster* is a highly tractable model organism with a wealth of genetic tools and reagents and is adaptable to numerous experimental modalities. Decades of research led to thorough understanding of its cell and developmental biology. Therefore, perturbations in different aspects of its development can be readily traced back to specific gene/s or pathway/s. These characteristics makes *Drosophila* a valuable tool to discover new or underappreciated functions of bacterial effectors, particularly outside the confounding effects of infection. This approach has been used to study effectors from *Helicobacter* (Reid et al., 2012) and *Chlamydia* (Shehat et al., 2021). *Drosophila*-derived cell lines have also been used to study the *Yersinia* effector YopJ (Paquette et al., 2012).

Tarp promotes F-actin bundling *in vitro* (Jiwani et al., 2013) though, prior to this work, this has not been demonstrated in a physiological context. It is, therefore, significant that Tarp induces a change in shape in wild-type adult mechanosensory bristles, since bristle shape is intimately linked to F-actin bundling during pupal development. Moreover, we showed that Tarp can partially rescue the morphology defect caused by loss of the endogenous bundler Singed/Fascin. This is a strong demonstration of Tarp’s F-actin bundling activity *in vivo*. We also believe that these phenotypic changes are primarily due to Tarp’s bundling activity rather than its actin polymerization activity. F-actin polymerization primarily occurs at the growing tip of the pupal bristle (DeRosier and Tilney, 2000), away from the mature F-actin bundles, where Tarp is localized. Last, increased F-actin polymerization in bristles manifest as forking or branching, a phenotype which was not observed upon Tarp expression (Hopmann and Miller, 2003).

We discovered a novel, competitive interaction between Tarp and Fascin during F-actin bundling. When simultaneously added to filamentous actin *in vitro*, Tarp outcompetes Fascin during bundling, relegating the endogenous bundler away from F-actin bundles. Competitive behavior between different F-actin bundlers has been documented. Two host bundlers, Fascin and alpha-Actinin, exclude each other from bundle formation due to a difference in F-actin packing mediated by the size of the cross-linking molecule (the alpha-Actinin molecule is long; Fascin is compact) (Winkelman et al., 2016). It is possible that the competition observed between Tarp (>150kDa) and Fascin (55kDA) occur *via* a similar mechanism. Alternatively, Tarp might have a higher bundling affinity compared to Fascin, outpacing Fascin’s ability to form F-actin bundles. Our *in vitro* finding is consistent with the observed *in vivo* displacement of *Drosophila* Singed from F-actin bundles when Tarp is present and supports a direct influence of Tarp on Singed localization *in vivo* rather than through some other cellular mechanism.

*In vivo*, Chlamydia infections occur at mucosal linings, whose epithelial cells are covered in microvilli on the apical face. Microvilli are short, hair-like membrane projections that are stabilized by crosslinked F-actin bundles, similar to *Drosophila* pupal bristles. A Chlamydia elementary body will interact and possible enter through this microvilli-rich cell surface, and early effectors such as Tarp may have to interact with this particular actin environment, perhaps through Tarp’s ability to compete with endogenous actin bundlers.

The validation of Tarp’s bundling activity *in vivo* and its ability to compete with endogenous host bundlers during F-actin bundle formation adds to the repertoire of molecular functions that Tarp can deploy during Chlamydia infection. Promoting F-actin polymerization at the site of entry is a major Chlamydia strategy during host cell invasion (Jewett et al., 2006; McKuen et al., 2017; Ghosh et al., 2020; Keb et al., 2021) but F-actin bundling itself has yet to be mechanistically implicated. Our finding raises the possibility that F-actin bundling and/or competition with host bundlers is a component of Tarp function in promoting Chlamydia infection.

In this study, we demonstrated physiological evidence of Tarp’s bundling activity *in vivo*. Furthermore, we discovered that Tarp inhibits Fascin’s innate ability to participate in bundle formation. This property is consistent with the *in vivo* observation of Singed/Fascin displacement upon Tarp expression in developing bristles. Lastly, this work establishes the utility of *Drosophila melanogaster* as a platform to discover new effector functions that do not readily manifest in an *in vitro* tissue culture system.

## Data Availability Statement

The raw data supporting the conclusions of this article will be made available by the authors, without undue reservation.

## Author Contributions

GA is responsible for the main concept and design of the study, with advice from TJ. GA and JK performed all Drosophila-related work. TJ performed the *in vitro* bundling assay. GA wrote the drafts of the manuscript. GA and TJ revised and approved the submitted version. All authors contributed to the article and approved the submitted version.

## Funding

This work is supported by grants from the National Institutes of Health, NIAID, R01AI139242 and R21AI148999 awarded to TJ.

## Conflict of Interest

The authors declare that the research was conducted in the absence of any commercial or financial relationships that could be construed as a potential conflict of interest.

## Publisher’s Note

All claims expressed in this article are solely those of the authors and do not necessarily represent those of their affiliated organizations, or those of the publisher, the editors and the reviewers. Any product that may be evaluated in this article, or claim that may be made by its manufacturer, is not guaranteed or endorsed by the publisher.
