# Supplementary material for: The Chlamydia trachomatis Early Effector Tarp Outcompetes Fascin in Forming F-Actin Bundles In Vivo
Source: Front Cell Infect Microbiol. 2022 Mar 1;12:811407. doi: 10.3389/fcimb.2022.811407 (PMC8921475; doi:10.3389/fcimb.2022.811407)
Supplement: Supplementary file 1 [file DataSheet_1.pdf]

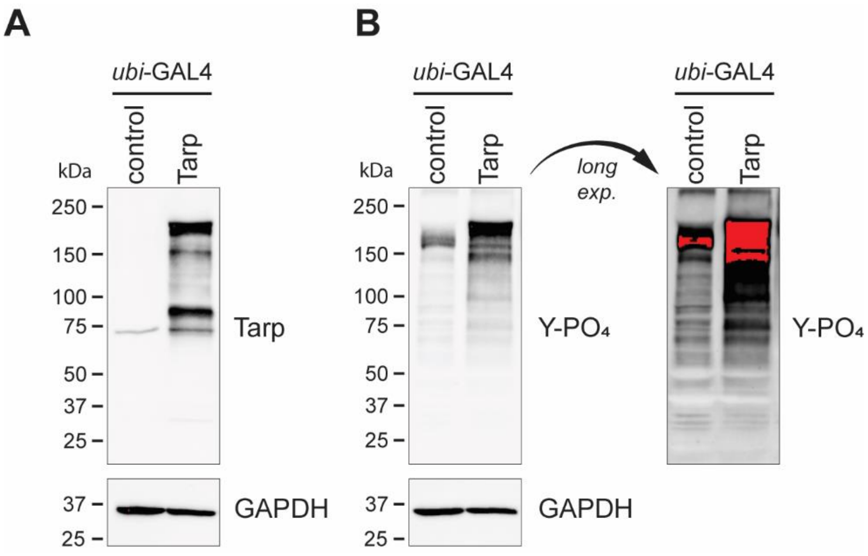

Figure S1. Ubiquitously expressed Tarp in *Drosophila* results in robust expression and phosphorylation. (A) Western blot of whole fly lysates showing Tarp expression in ubi-GAL4/UAS-Tarp flies but absent in ubi-GAL4/UAS-GFP control flies. (B) An identical blot was probed for tyrosine phosphorylation, showing strong tyrosine-phosphorylated bands that correspond to Tarp bands. Overexposure of the blot reveals endogenous tyrosine-phosphorylated proteins in control and Tarp-expressing fly lysates (red color indicates oversaturated pixels). GAPDH was used as loading control.

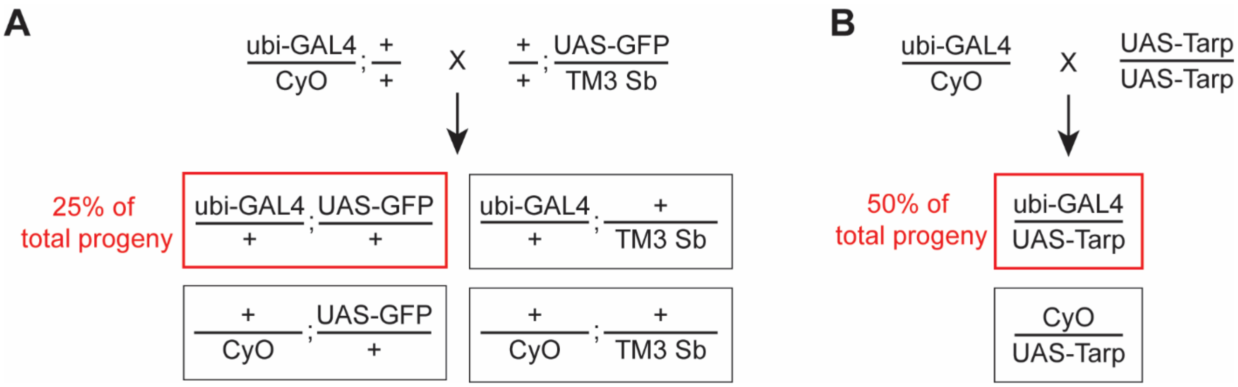

Figure S2. Crossing schemes to determine the impact of ubiquitous Tarp expression on adult viability. (A) ubi-GAL4/CyO flies were cross to UAS-GFP/TM3 Sb flies to generate control flies that ubiquitously express GFP. By Mendelian genetics, ubi-GAL4>UAS-GFP flies is expected to comprise 25% of the total adult progeny. (B) ubi-GAL4/CyO lies were crossed to UAS-Tarp homozygous flies to generate ubi-GAL4/UAS-Tarp flies, which drive Tarp expression throughout the whole animal. By Mendelian genetics, ubi-GAL4>UAS-Tarp flies are expected to comprise 50% of the total adult progeny if no lethality is observed.

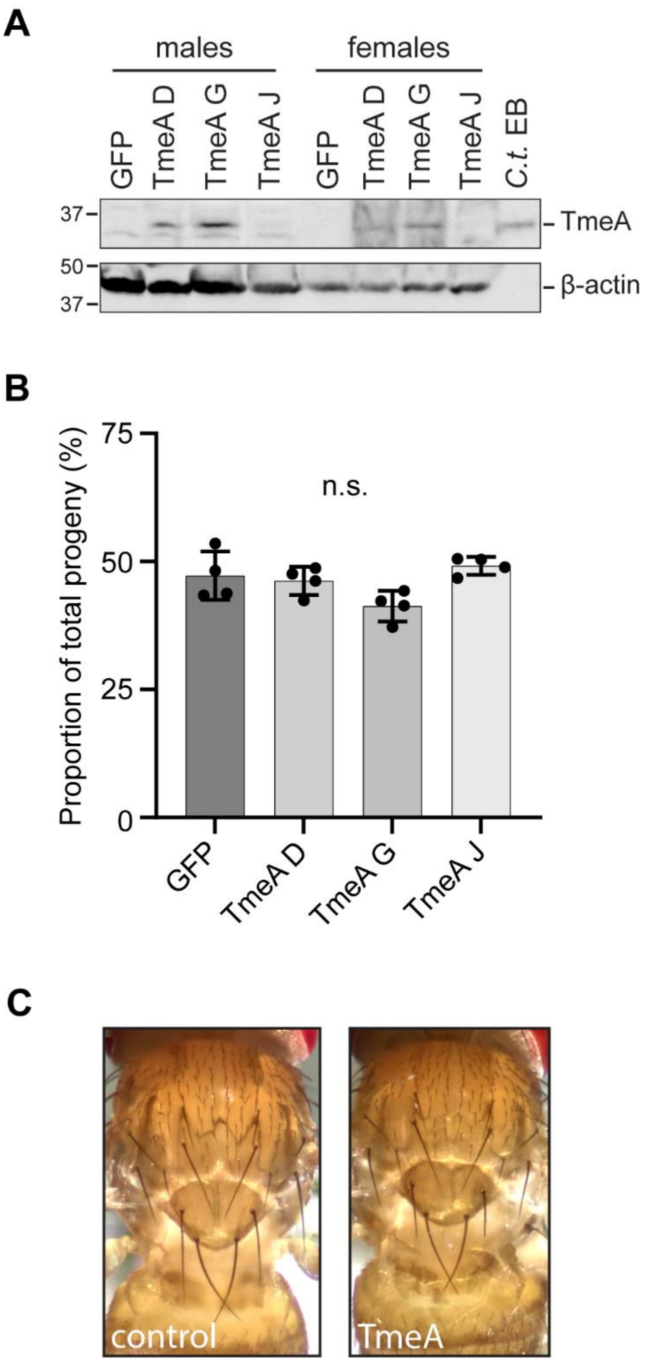

Figure S3. Ubiquitous expression of the *C. trachomatis* early effector TmeA does not impair fly viability and bristle morphology. (A) Ubi-GAL4 flies were crossed to UAS-GFP or different UAS-TmeA transgenic lines (D, G, and J). Whole fly lysates of ubi>GFP and ubi>TmeA were analyzed by Western blot for the expression of TmeA. Protein sample from *C.trachomatis* elementary bodies was used as positive control for TmeA expression. Actin serves as fly lysate loading control. (B) The proportion of flies that ubiquitously express GFP or TmeA within the total progeny was measured. There

was no statistical difference observed between the proportion of ubi>GFP and ubi>TmeA flies (Kruskal-Wallis test with Dunn’s multiple comparisons). The expected ratio of ubi>GFP and ubi>TmeA flies is 50% by Mendelian genetics. (C) pnr-GAL4 was crossed to UAS-GFP (control) or UAS-TmeA flies to generate flies that drive transgene expression along the thorax midline. The macrochaetes along the midline for either genotype have no abnormal curvature.

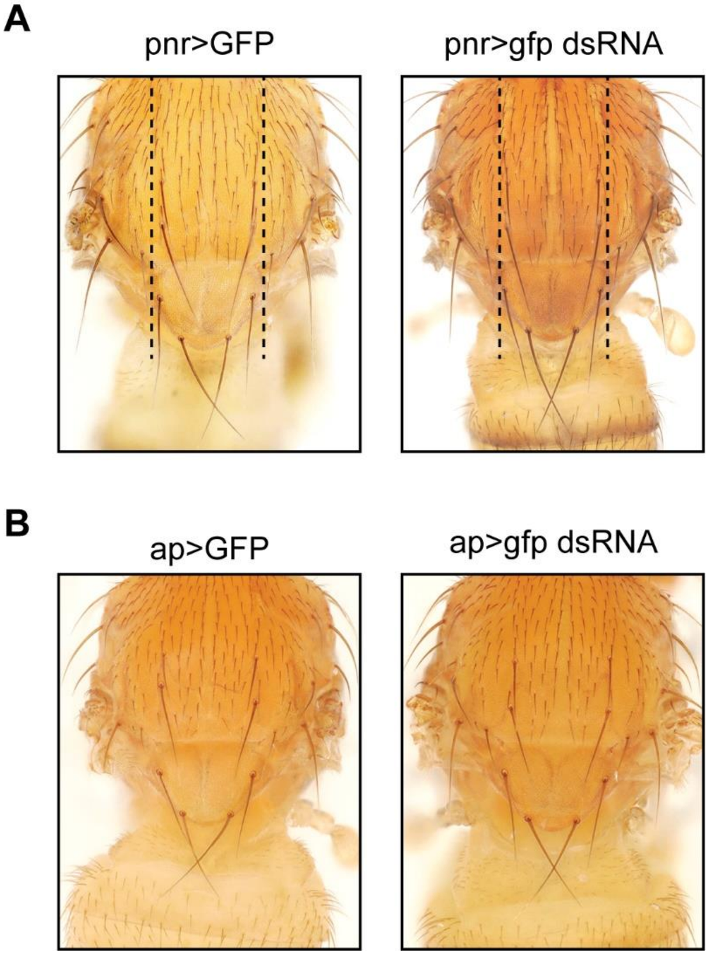

Figure S4. Normal bristle morphology upon engagement of the RNAi machinery in the dorsal thorax. Images of the adult dorsal thorax from flies expressing double-stranded RNA against GFP, a non-endogenous target, using (A) pnr-GAL4 or (B) ap-GAL4 drivers. Dashed lines represent the thorax midline expression domain of pnr-GAL4. ap-GAL4 expresses broadly throughout the thorax. GFP expression serves as control.

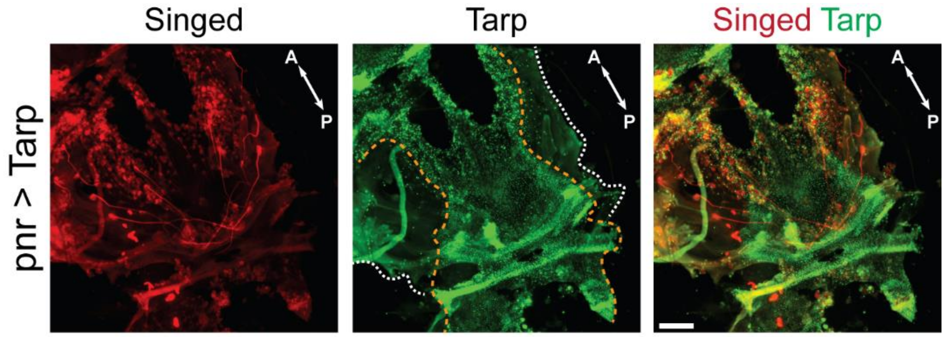

Figure S5. Dissected dorsal pupal pelt that encompass the developing thorax region displaying Tarp expression. The dorsal pelt of a UAS-Tarp/+;pnr-GAL4/+ (pnr>Tarp) pupa was dissected and immunostained for Singed (red) and Tarp (green). Tarp expression is enriched along the midline of the dorsal pupal pelt (region bound by dashed lines). Pupal bristles have high Singed protein levels. Anterior (A) and posterior (P) directions are indicated. Scale bar is 100μm.
